# The Emerging Role of Polyethylene Glycol-water (PEG-H_2_O) as the Benign Mixed Solvent System in Organic Synthesis

**DOI:** 10.2174/0115701794284081240206043435

**Published:** 2024-03-12

**Authors:** Chitteti Divyavani, Pannala Padmaja, Pedavenkatagari Narayana Reddy

**Affiliations:** 1 Department of Chemistry, Sri Padmavathi Women’s Degree & PG College, Tirupati, Andhra Pradesh, India;; 2 Centre for Semio Chemicals, CSIR-Indian Institute of Chemical Technology, Hyderabad, India;; 3 Department of Chemistry, School of Science, GITAM Deemed to be University, Hyderabad, India

**Keywords:** Polyethylene glycol, water, aqueous biphasic solvent systems, multicomponent reactions, heterocyclic compounds, green chemistry

## Abstract

Polyethylene glycol (PEG) has become a popular solvent and green catalyst for a variety of chemical reactions. It is a stable and biodegradable polymeric catalyst used in organic synthesis because it may be recycled numerous times without significantly losing its catalytic activity. Recently, the use of PEG-H_2_O mixed solvent systems in organic synthesis has gained popularity.

This article presents an overview of PEG-H_2_O solvent system-mediated organic reactions, with a main focus on the importance of the solvent system. The study also focuses on recent developments in the PEG-H_2_O solvent system-mediated synthesis of a number of heterocyclic compounds.

Important characteristics of these PEG-H_2_O solvent systems include high atom economies, gentle reaction conditions, faster reaction rates, readily isolated side products and high yields. Results from various reactions showed that the choice of proper ratio of PEG: H_2_O solvent system plays a key role in product yields.

## 1 INTRODUCTION

Green chemistry is a pioneering field that generally reports intrinsic atom economy, energy savings, waste reduction, simple workups, and the avoidance of dangerous compounds. In order to achieve the Sustainable Development Goals, green chemistry, which focuses on process design to limit the usage and synthesis of harmful substances, is essential [1, 2]. In recent years, there has been a surge of interest in both commercial and academic research in the creation of environmentally friendly chemical synthesis [3, 4]. One of the primary challenges has been the development of cost-effective and environmentally favorable catalytic systems. Organic solvents are commonly utilized in numerous chemical processes and have posed a serious environmental danger due to their toxic nature. As a result, the scientific community is looking for alternatives to toxic, dangerous, volatile, and difficult-to-recover organic solvents. To address these issues, the volatile and poisonous organic solvents used as a reaction medium were replaced with environmentally friendly alternatives. The common solvents now in use have a number of issues, including the fact that they are dangerous, combustible, poisonous, accumulating, and non-biodegradable. Due to their safety, non-toxicity, affordability, sustainability, and recyclable nature, green organic solvents are becoming increasingly popular in modern synthetic protocols. The most widely used green solvents include water [5, 6], gas-expanded liquids [7], liquid polymers [8, 9], supercritical fluids [10, 11], ionic liquids (ILs) [12-15] and deep eutectic solvents (DESs) [16-19].

Polyethylene glycol (PEG) is the most basic non-ionic synthetic polymer, with applications ranging from chemical technology to pharmaceutical and medical applications. Because of its outstanding structural flexibility, amphiphilicity, lack of steric hindrances, and high hydration capacity, it is recognized as a promising solvent system [20-34]. The inexpensive, recyclable nature, non-volatility, low flammability, low toxicity, simplicity of degradation, and high thermal stability are other distinctive characteristics of PEG [35]. It is employed in organic synthesis as a phase transfer catalyst, generally substituting costly and hazardous catalysts, as well as a safe and effective reaction medium. It is very helpful as a reaction medium for different organic processes because of its ability to dissolve a wide variety of compounds [36-40]. Contrarily, water is the most widely available solvent and the most economical fluid for chemical synthesis. Most likely, water is a benign, non-explosive, or nonflammable solvent. Furthermore, the highest specific heat capacity of water makes exothermic reactions easier to handle [35, 41-44].

Additionally, organic synthesis has become more efficient generally, and there is a significant chance that organic emissions to the environment can be decreased. Aqueous-based solvent systems, such as those based on water, may become a more prevalent option to replace conventional solvents in synthetic chemistry.

Multi-component reactions (MCRs) in water have been shown to be powerful tools in the search for developing libraries of medicinal scaffolds as well as for the requirements of green chemistry. As a result, scientists are focusing increasingly on ecologically friendly synthesis in the development of MCRs, such as metal-free catalysts, eco-friendly solvents, renewable resources, and waste minimization. The use of reusable catalysts for multi-component reactions in water has recently been established, and it is related to green chemistry procedures for MCRs. The use of PEG-H_2_O mixed solvent systems in MCRs has gained popularity recently. For a number of reasons, water is an excellent choice for a PEG solvent media [45-48]. Because PEGs quickly absorb water from the ambient, water is the principal contaminant. PEG forms hydrogen bonds both within and between molecules, increasing its solubility in water [49]. Water and lower molecular weight PEGs are completely miscible at normal temperatures. However, liquid-liquid phase separation happens at high temperatures. Aqueous biphasic systems (ABSs) are the name given to these systems, which have been utilized to recover and purify organic compounds. After the completion of the reaction, water is typically added to PEG to force the product to precipitate. It is well known that adding PEG as a co-solvent to water reduces the apparent polarity of the aqueous solution. PEG is thought to aid in bringing the aqueous and organic reagents together by acting as a phase transition catalyst. Utilizing a PEG-H_2_O mixed solvent can have inherent advantages, such as accelerating reaction rates or making side products easier to identify. Their low toxicity, low volatility, and biodegradability are major environmental features that are especially enticing when combined with their low cost as a bulk commodity chemical. Aqueous PEG solutions can also regularly replace phase transfer catalysts (PTCs), which are expensive. In a variety of organic processes, PEG-H_2_O binary solvent media are frequently utilized. Many organic reactions are developed in an aqueous medium in the presence of PEG, which solubilizes organic components in water. The use of PEG aqueous reaction media has only been briefly discussed in a few reviews [36, 50]. However, none of these reviews have specifically addressed how organic transformations are mediated by PEG-H_2_O mixed solvent systems. Here, we provide a thorough overview and analysis of PEG-H_2_O reaction media's function in a range of organic chemical reactions. In comparison to other solvent systems, the importance of the PEG-H_2_O solvent system is also explored. The mechanisms of key reactions are discussed.

## 2 NAMED REACTIONS

### 2.1 Ugi Reaction

**Scheme 1:**
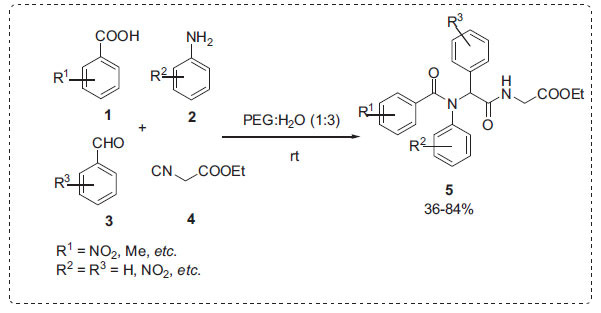
Ugi reaction in PEG-H_2_O.

The Ugi four-component reaction was created to produce natural compounds and pharmaceuticals with high yields and diastereoselectivities [51, 52]. Niu *et al*. reported a Ugi reaction between a carboxylic acid **1**, an amine **2**, a carbonyl compound **3** and an isocyanide **4** to afford bisamides **5** in PEG-H_2_O as a solvent under mild conditions (Scheme **1**) [53]. In this approach, reactants with low water solubility could be used at large concentrations in an aqueous media with no side effects. No product was obtained in pure PEG. However, the yield was significantly improved in PEG-H_2_O. Because of the hydrophobic effect and hydrogen bonding, the PEG-H_2_O (1:3) medium can efficiently accelerate the process. Since the product was insoluble in a PEG-H_2_O solvent medium, its isolation was made easier by filtration.

### 2.2 Knoevenagel Condensation Reaction

**Scheme 2:**
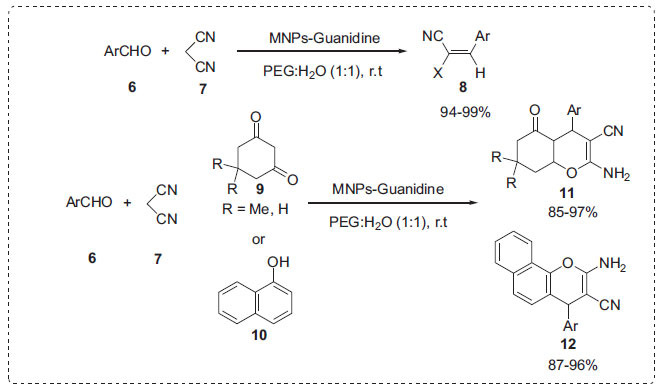
Knoevenagel condensation for the synthesis of chromenes in PEG-H_2_O.

Emil Knoevenagel discovered Knoevenagel condensation, which is a vital reaction for the preparation of *α*,*β*-unsaturated compounds [54]. In an intriguing piece of work, Rostami *et al*. studied the application of MNPs-guanidine nanocatalyst for the Knoevenagel condensation reaction of aromatic aldehydes **6** with malononitrile **7** in PEG-H_2_O (1:1) at room temperature to afford alkenes **8** (Scheme **2**) [55]. The same catalyst was useful for the synthesis of 2-amino-4*H*-chromenes **11** and 2-amino-4*H*-benzo[*h*]chromenes **12** by the reaction of aldehydes **6**, malononitrile **7** and cyclic 1,3-dicarbonyl compound **9** or *α*-naphthol **10**. The supported catalyst could be easily retrieved from the reaction mixture and reused numerous times with the help of an external magnet.

### 2.3 Mannich Reaction

**Scheme 3:**
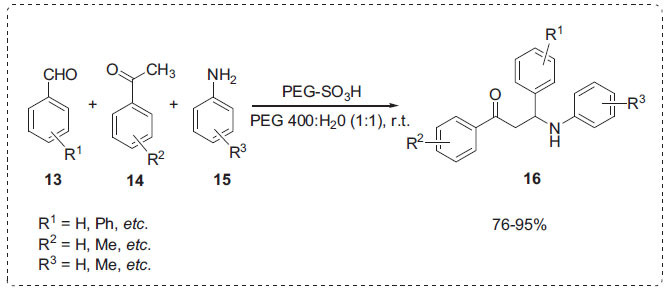
Mannich reaction in PEG-H_2_O.

The Mannich reaction is a key carbon-carbon bond formation reaction in the synthesis of *β*-amino carbonyl compounds [56]. Wang *et al*. applied sulfuric acid-modified polyethylene glycol 6000 (PEG-OSO_3_H) as a catalyst for the synthesis of *β*-amino carbonyl compounds **16** *via* the Mannich reaction between aldehydes **13**, aromatic ketones **14** and aromatic amines **15** at room temperature in PEG 400-H_2_O solvent (Scheme **3**) [57]. The 1:1 solvent mixture of PEG 400-H_2_O affords the highest product yields. The catalyst was recovered and reused seven times. The reactions were conducted quickly under mild reaction conditions, with yields.

### 2.4 Michael's Addition Reaction

The Michael reaction, also known as the Michael 1,4 addition, is a reaction that occurs between a Michael donor and a Michael acceptor to produce a Michael adduct by forming a carbon-carbon bond [58].

**Scheme 4:**
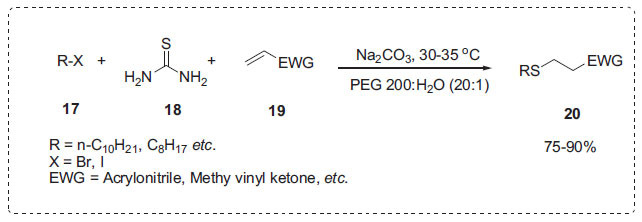
Thia-Michael reaction in PEG-H_2_O.

Firouzabadi *et al*. devised an odourless synthesis of thia-Michael adducts **20** by reacting different organic halides **17**, electron-deficient alkenes **19**, and thiourea **18** in PEG 200-H_2_O at 30-35°C with sodium carbonate (Scheme **4**) [59]. To highlight the important role of PEG in this reaction, the authors performed the identical reaction in water as well as an aqueous solution of *β*-cyclodextrin. These reactions, however, failed, and the starting materials were separated intact from the reaction mixtures. This protocol is very beneficial for large-scale operations.

**Scheme 5:**
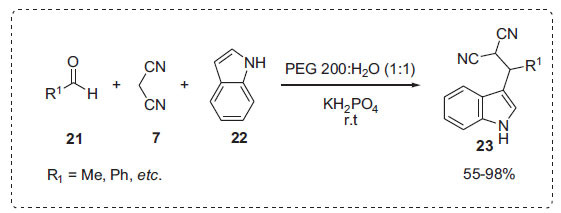
Synthesis of 3-indole derivatives.

Following previous reactions, Wang *et al*. reported the synthesis of 3-indole derivatives **23** by the three-component reaction of indoles **22**, malononitrile **7** and aldehydes **21** in water, which was promoted by PEG-200-H_2_O (Scheme **5**) [60]. A low isolated yield of the desired product was reported in pure PEG-200 or pure water. However, when the water-to-PEG-200 mass ratio was adjusted to 1:1, the product yield increased significantly. The reaction was repeated three times and observed same yields.

### 2.5 Strecker Reaction

**Scheme 6:**
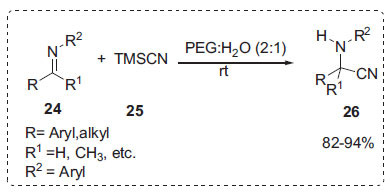
Strecker synthesis of *α*-aminonitriles in PEG/H_2_O.

Strecker reaction is a simple technique for producing *α*-aminonitriles, which are required intermediates to produce amino acids [61, 62]. In this regard, Reddy *et al*. discovered that PEG-H_2_O medium was an inexpensive, non-toxic, and environmentally friendly reaction medium for the nucleophilic addition of trimethylsilyl cyanide **25** (TMSCN) to imines **24** to give excellent yields of *α*-aminonitriles **26** (Scheme **6**) [63]. PEG in water was chosen as the reaction medium for three reasons: (i) it is environmentally friendly; (ii) PEG can precipitate and be recycled during the work-up; and (iii) PEG catalyses the cyanide nucleophilic addition to the imine carbon by increasing its electrophilicity through hydrogen bonding by its hydroxyl group with the nitrogen of the imine, with water acting as a proton source. This method works on a variety of substrates and does not require the use of an acid or basic catalyst. PEG is recyclable and reusable.

### 2.6 Cross-coupling Reactions

PEGs have been used in many cross-coupling reactions due to possible recycling of catalyst and reaction medium.

#### 2.6.1 Suzuki-miyaura Reaction

**Scheme 7:**
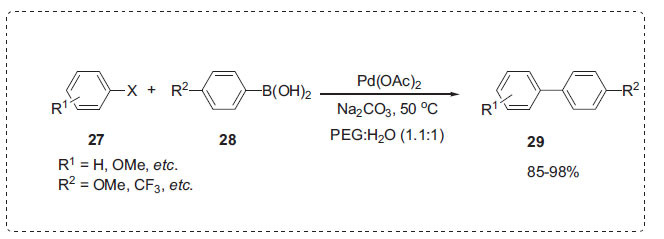
Suzuki reaction in PEG-H_2_O.

The Suzuki coupling reaction is a versatile method for creating unsymmetrical biaryls [64, 65]. Zhang and co-workers explored the influence of PEG on the Suzuki reaction in water [66]. The authors used Pd(OAc)_2_, in combination with PEG as a catalyst for the Suzuki coupling reaction of aryl halides **27** and boronic acids **28** in water to afford the biaryls **29** (Scheme **7**). The impact of the PEG-H_2_O system with different organic solvents was investigated by the authors. According to these findings, the solvent has a significant impact on the reaction, and water is an effective solvent for the Suzuki reaction when PEG is present. The reaction can be carried out at 50°C with high yields without the need of phosphine ligand or a microwave. In terms of product yields, PEG 2000 was superior to PEG 600 and PEG 1000. Diethyl ether is utilized to separate the products, and both the catalyst and solvent can be reused without losing much activity.

**Scheme 8:**
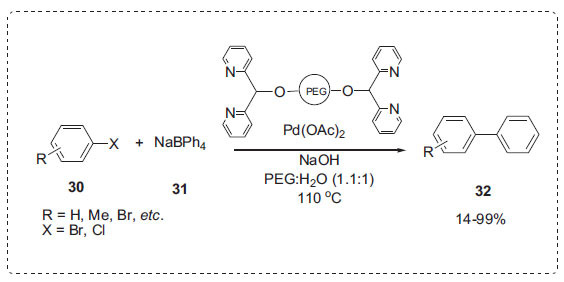
Suzuki-type reaction PEG-H_2_O.

In a different study, Gao *et al*. synthesized an air- and water-stable PEG-supported bidentate nitrogen ligand for the palladium-catalyzed Suzuki-type reaction of aryl halides **30** with sodium tetraphenylborate **31** in aqueous PEG to produce biaryls **32** (Scheme **8**) [67]. A wide range of aryl halides, including aryl bromides and chlorides, were easily coupled to sodium tetraphenylborate. In addition, there was no discernible loss of catalytic activity for the Suzuki process after recycling and reusing the homogeneous catalyst system five to six times. The optimum base for the reaction was sodium hydroxide.

**Scheme 9:**
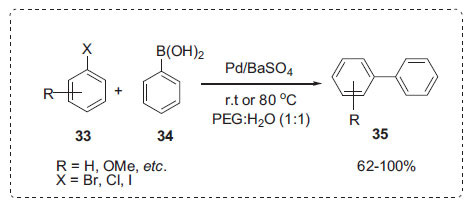
Synthesis of biaryls.

According to De Souza *et al*., the Suzuki-Miyaura reactions between halobenzenes **33** and boronic acids **34** were conducted using Pd/BaSO_4_ as a catalyst in PEG-H_2_O solution without any ligand to afford biaryls **35** (Scheme **9**) [68]. When a reaction was carried out at room temperature, the addition of water to PEG 300 had a dramatic effect, yielding 100%. The catalytic system and reaction medium were reused without any loss of activity.

**Scheme 10:**
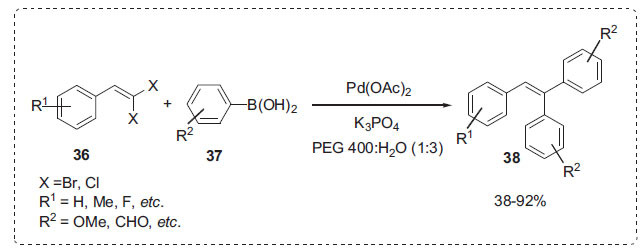
Synthesis of triarylethenes.

In a different study, Zhu *et al*. reported highly selective Pd-catalyzed double Suzuki-Miyaura coupling reactions of 1,1-dibromo-1-alkenes **36** with various arylboronic acids **37** in PEG aqueous solution under mild conditions to afford the triarylethenes **38** (Scheme **10**) [69]. It was discovered that using PEG and water as a combined solvent produced better product yields than using PEG or water alone. The authors hypothesised that the active catalytic species could be palladium nanoparticles produced *in situ* in PEG. For the synthesis of triarylethene-based compounds, this process is straightforward, cost-effective, and practical.

#### 2.6.2 Hiyama Coupling Reaction

**Scheme 11:**
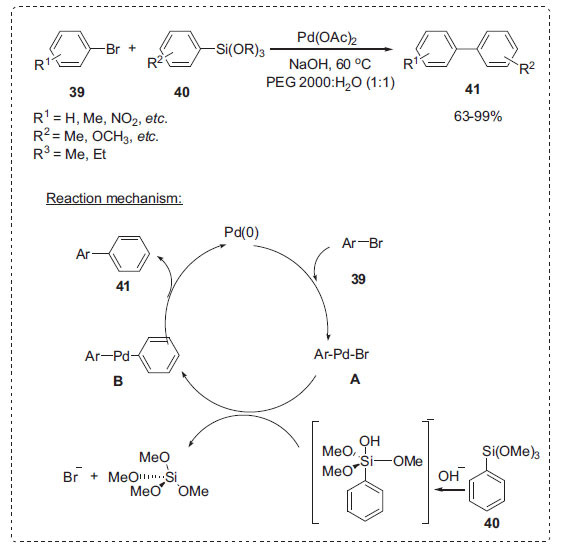
Synthesis of biaryls in PEG-H_2_O.

Hiyama cross-coupling is a useful synthetic organic chemistry method for forming carbon-carbon bonds [70]. Shi *et al*. utilized the PEG-H_2_O solvent system as an alternative reaction medium for the cross-coupling reaction between the aryl bromides **39** and arylsiloxanes **40** with Pd(OAc)_2_ as a catalyst to afford biaryls **41** (Scheme **11**) [71]. It was discovered that PEG 2000 in water significantly increased reactivity by shortening reaction durations and greatly enhancing yields under mild reaction conditions. However, the reactions were not successful either in the neat PEG or in water alone. As expected, the coupling reaction was sluggish in PEG solvent despite the fact that the presence of water improved the sodium hydroxide's solubility in this catalytic system. On the other hand, PEG significantly increased the solubility of the organic substrates in water, which also increased the rate of the reaction. Ethyl ether extraction made it simple to separate the product, and the catalytic system can be reused eight times effectively. The following mechanism is proposed in the sodium hydroxide aqueous solution (Scheme **11**). The initial oxidative addition of aryl bromide **39** to Pd(0) generates the arylpalladium intermediate **A**, which reacts with the pentavalent silicate formed *in situ* in sodium hydroxide solution to afford the (aryl)(aryl)- palladium(II) species **B**. The subsequent reductive elimination liberates the biaryl product **41** with the regeneration of the active Pd(0) to complete the catalytic cycle.

#### 2.6.3 Barluenga-Valdés Cross-coupling Reaction

**Scheme 12:**
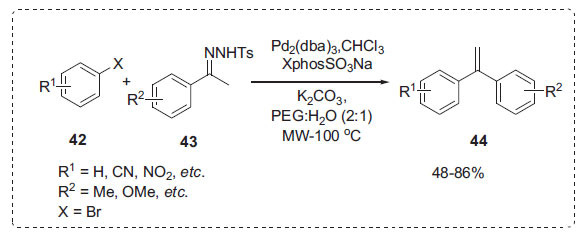
Synthesis of 1,1-diarylethylene compounds.

The groups of Barluenga and Valdés created a Pd-catalyzed Csp^2^-Csp^2^ bond-forming process that uses *N*-tosylhydrazones (NTH) produced from ketones as a nucleophilic partner and organic halides as a catalytic partner [72]. For the synthesis of 1,1-diarylethylenes **44**, Lamaa *et al.* created a green Barluenga-Valdés cross-coupling reaction of aryl halides **42** and *N*-tosylhydrazones **43** employing a novel catalytic system based on Pd/Xphos−SO_3_Na or Pd/MeDavephos-CF_3_SO_3_ in PEG/H_2_O under microwave irradiation (Scheme **12**) [73]. The PEG-400:H_2_O (2:1) solvent in conjunction with microwave irradiation produced the greatest yield. K_2_CO_3_ as a base proved to be an excellent choice for this reaction. It was revealed that performing the reaction simply in PEG-400 or only in water lowered the yield substantially. The catalytic system was recycled nine times without substantial activity loss.

#### 2.6.4 Sonogashira Coupling Reaction

**Scheme 13:**
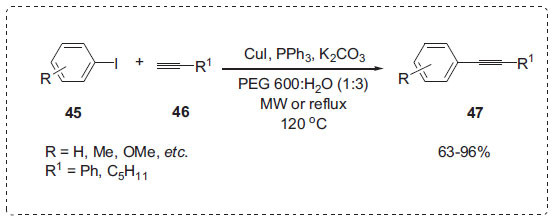
Cross-coupling of aryl iodides with alkynes.

The Sonogashira reaction has proven to be one of the most efficient and straightforward methods for forming carbon (Sp2)-carbon (sp) bonds [74]. In this synthesis field, Chen *et al.* devised an efficient technique for the copper-catalyzed Sonogashira coupling of aryl iodides **45** with terminal acetylenes **46** in PEG-H_2_O to yield alkynes **47** (Scheme **13**) [75]. The best outcome was achieved utilizing a 1:3 PEG 600:H_2_O solvent mixture with CuI as a catalyst and K_2_CO_3_ as a base. PEG served as a co-solvent and a phase-transfer catalyst (PTC), and factors such as the PEG molecular weight and chain end effects might affect the phase catalytic activity. Both electron withdrawing and electron releasing aryl iodides were participated under microwave heating or reflux in oil bath to afford the corresponding products in good yields.

#### 2.6.5 Other Cross-coupling Reactions

**Scheme 14:**
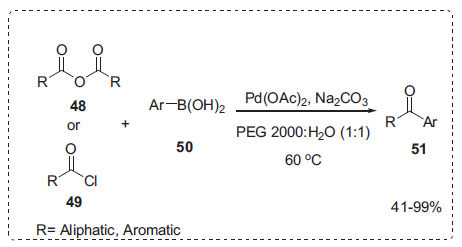
Cross-coupling of acid anhydrides or acid chlorides with arylboronic acids.

Inspired by the above methods, Xin *et al*. developed the coupling reaction of aryl boronic acids **50** with carboxylic anhydride **48** or acyl chloride **49** in the presence of Pd(OAc)_2_-H_2_O-PEG homogeneous aqueous catalytic system to afford ketones **51** without the use of phosphine ligands (Scheme **14**) [76]. The addition of PEG led to a rapid increase in yield in the aqueous media. The optimal ratio of PEG-H_2_O was 1:1. Among the different PEGs tested, PEG 2000 showed the best reactivity. When the various bases were screened, Na_2_CO_3_ and K_2_CO_3_ resulted in a good yield. PEG 2000 has a solubility of about 60% in water at 20°C. PEG 2000, on the other hand, was insoluble in diethyl ether; however, the product was easily separated by extraction with diethyl ether.

**Scheme 15:**
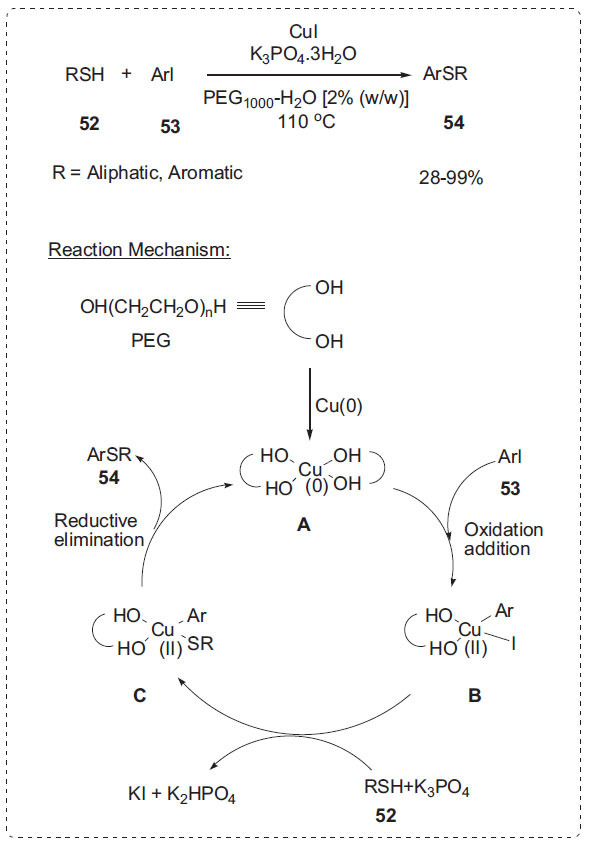
Synthesis of aryl sulfides.

A new procedure for coupling aryl iodides **53** with thiophenols or alkane thiols **52** using the CuI-PEG-H_2_O system was published by She *et al*. in a different study (Scheme **15**) [29]. The aryl sulphides **54** were produced in excellent yields in an aqueous solution containing only 2% PEG 1000 without any other additives or ligands. The extraction using diethyl ether or petroleum ether makes it simple to isolate the products. The catalytic system is both inexpensive and environmentally benign. The possible mechanism for the copper-catalyzed *S*-arylation of thiols is depicted in Scheme **15**. Cu(I) or Cu(II) was reduced by PEG to form Cu(0), which conjugates with PEG to form a reactive species **A**. The subsequent oxidative addition of the **A** with aryl iodides **53** leads to the intermediate **B**. In the presence of base, thiols **52** react with **B** readily to afford complex **C**, which undergoes a reductive elimination to provide the target product **54** and to regenerate the reactive species **A**.

**Scheme 16:**
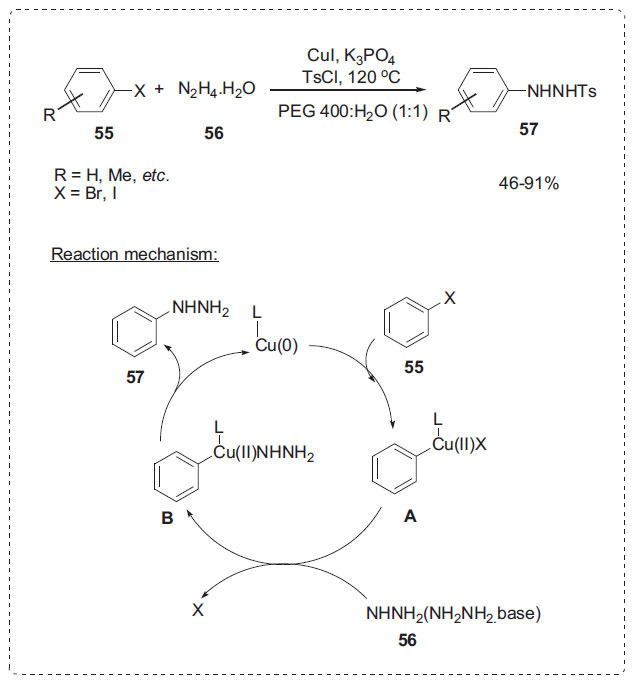
Synthesis of arylhydrazines.

In continuation, Chen *et al.* developed an effective and convenient technique for producing aryl hydrazines **57** by copper-catalyzed cross-coupling of aryl halides **55** with hydrazine **56** in aqueous PEG-400 using copper iodide and K_3_PO_4_ (Scheme **16**) [77]. This procedure provides hydrazines with good to outstanding yields from both electron-deficient and electron-rich aryl iodides and bromides, as well as sterically hindered substrates. The mechanism is illustrated in Scheme **16**. The oxidative addition **A** of a Cu(0) species to the carbon-halogen bond should initially take place, followed by the halogene-hydrazine exchange reaction and reductive elimination of the Cu species **B**.

### 2.7 C-H Activation Reactions

**Scheme 17:**
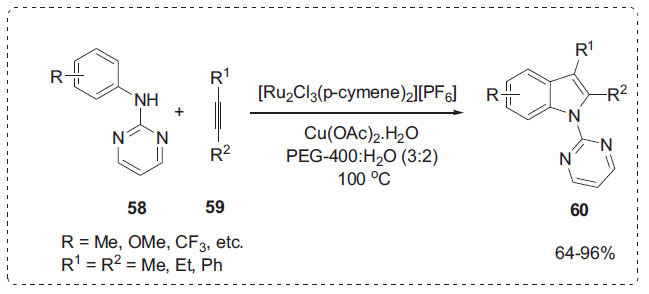
Synthesis of indole derivatives.

Transition-metal-catalyzed C-H bond activation has opened up a new avenue for the synthesis of a wide range of heterocycles [78, 79]. In this context, Liao *et al*. developed oxidative annulation of alkynes **59** by *N*-2-pyrimidyl-substituted anilines **58** bearing a removable directing group in a mixture of PEG-400 and water catalyzed by [Ru_2_Cl_3_(*p*-cymene)_2_][PF_6_] (Scheme **17**) [80]. The reaction could be carried out at 100°C with the oxidant Cu(OAc)_2_·H_2_O, generating a variety of indole derivatives **60** in good yields. The reaction moved slowly when PEG-400 was utilized as the solvent. However, using a solvent mixture of PEG-400 and H_2_O could increase product yield. The products were easily isolated by extraction with petroleum ether. Moreover, both [Ru_2_Cl_3_(*p*-cymene)_2_][PF_6_] and Cu(OAc)_2_ in the PEG-400/H_2_O system could be recycled and reused six times without loss of catalytic activity.

**Scheme 18:**
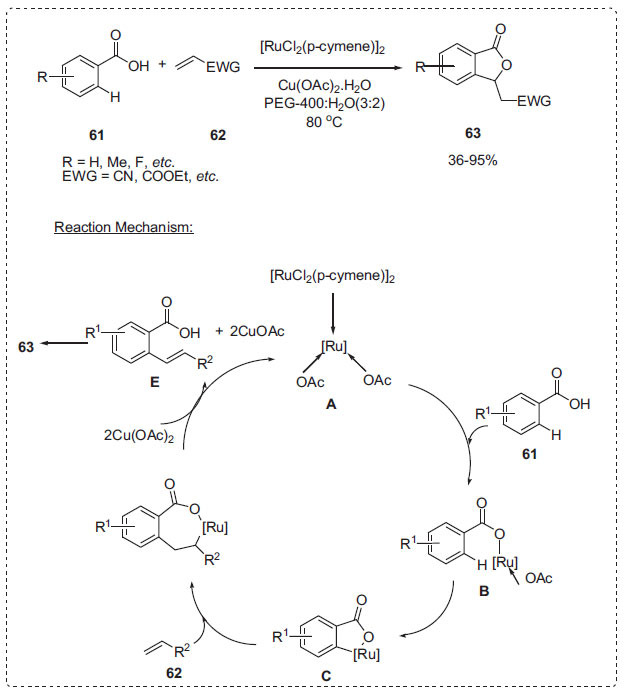
Synthesis of phthalides.

Similarly, Zhao *et al*. used a [RuCl_2_(*p*-cymene)_2_] [PF_6_]/Cu(OAc)_2_/PEG-400/H_2_O system as a highly efficient and reusable catalytic medium for the cross-dehydrogenative C-H bond alkenylation process between benzoic acids **61** and alkenes **62** (Scheme **18**) [81]. The reaction could be carried out at 80°C using Cu(OAc)_2._H_2_O as the oxidant to produce a wide range of phthalide derivatives **63** in good to excellent yields. When PEG-400 was utilized as the sole solvent, the reaction clearly moved slowly. However, a PEG-400/H_2_O mixture was found to be more effective than PEG-400 alone. More notably, in the PEG-400/H_2_O system, both [RuCl_2_(*p*-cymene)]_2_ and Cu(OAc)_2_ could be efficiently recycled and reused six times without losing catalytic activity. A plausible mechanism for the ruthenium-catalyzed oxidative C-H bond alkenylation reaction of benzoic acids **61** with alkenes **62** is illustrated in Scheme **18**. First, the reaction of [RuCl_2_(*p*-cymene)] with acetate generates *in situ* ruthenium(II) diacetate complex **A**. Coordination of the carboxyl oxygen of **61** to the ruthenium(II) diacetate complex **A** with the liberation of AcOH gives a ruthenium(II) benzoate **B**. Subsequent *ortho*-C-H bond ruthenation to form a ruthenacycle intermediate **C**, alkene **62** insertion, and *β*-hydride elimination successively occur to produce an olefinated intermediate **E**. The latter undergoes an intramolecular oxa-Michael addition reaction to afford the desired phthalide 6**3**. After the release of intermediate **E**, the resulting [Ru(0)] species may be oxidized in the presence of Cu(OAc)_2_ to regenerate ruthenium(II) diacetate complex **A**.

### 2.8 Hydrogenations

**Scheme 19:**
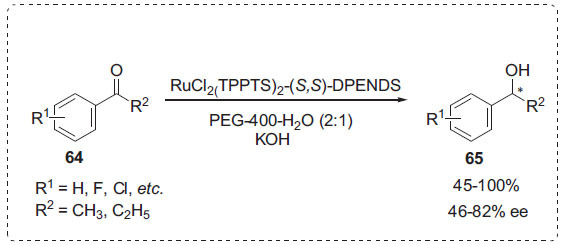
Synthesis of chiral alcohols.

Asymmetric hydrogenation is an effective approach for producing enantiomerically pure compounds [82]. PEGs, in combination with organic co-solvents have been described as asymmetric hydrogenation reaction medium [83, 84]. In this regard, Qin *et al*. developed asymmetric hydrogenations of aromatic ketones **64** catalyzed by a ruthenium achiral monophosphine complex RuCl_2_ (TPPTS)_2_ [TPPTS:P(m-C_6_H_4_SO_3_Na)_3_] modified by (S,S)-DPENDS [disodium salt of sulfonated (S,S)-1,2-diphenyl-1,2-ethylene-diamine] to provide the chiral alcohols **65** using PEG-400/H_2_O as a green and recyclable reaction medium (Scheme **19**) [85]. According to the findings, adding appropriate amounts of water can enhance the ee value while also improving the catalyst's solubility and the concentration of H_2_ in PEG-400.

Extraction using *n*-hexane efficiently separates the resultant compounds from the catalyst. The catalyst immobilized in PEG-400-H_2_O not only has high activity and enantioselectivity but it can also be recycled and reused without losing activity or enantioselectivity.

**Scheme 20:**
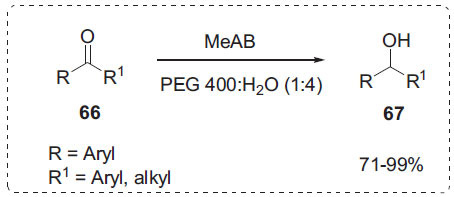
Hydrogenation of aromatic ketones.

In another interesting work, Duan *et al*. demonstrated chemoselective hydrogenation of various aldehydes and ketones **66** with methylamine borane (MeAB) in neat water and PEG to afford alcohols **67** (Scheme **20**) [86]. This hydrogenation method is applicable to a wide range of aliphatic,aromatic, -unsaturated, and heterocyclic aldehydes and ketones. The solubility is important for the reaction rate. PEG 400 enhances reactant solubility, resulting in a greater interfacial area and lower mass transfer resistance.

### 2.9 *N*-allylation

**Scheme 21:**
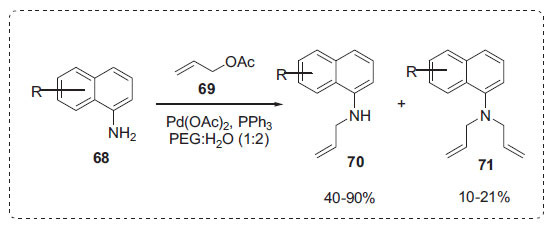
*N*-allylation of 1-aminonapthalene.

Allylamines are pivotal building blocks for the synthesis of key intermediates in the total synthesis of natural products [87]. Shih *et al*. described an environmentally friendly, effi-cient catalytic method involving palladium and ligands in a PEG 4000-water system that resulted in *N*-allylation (Scheme **21**) [88]. The addition of PEG 4000 led to a rapid increase in the activity of palladium-catalyzed allylic amination of allylic acetates **69** with aminonaphthalenes **68**. The corresponding mono and di-substituted *N*-allylic aminonaphthalenes **70**, **71** were obtained in good yields. PEG 4000 outperformed all other PEGs. Good to exceptional yields were obtained from both electron-rich and electron-withdrawing groups. The PEG 4000/H_2_O system has three significant advantages: all reactions are performed in water, no additional additives are required other than PEG, and reaction temperatures are frequently less than 100°C.

**Scheme 22:**
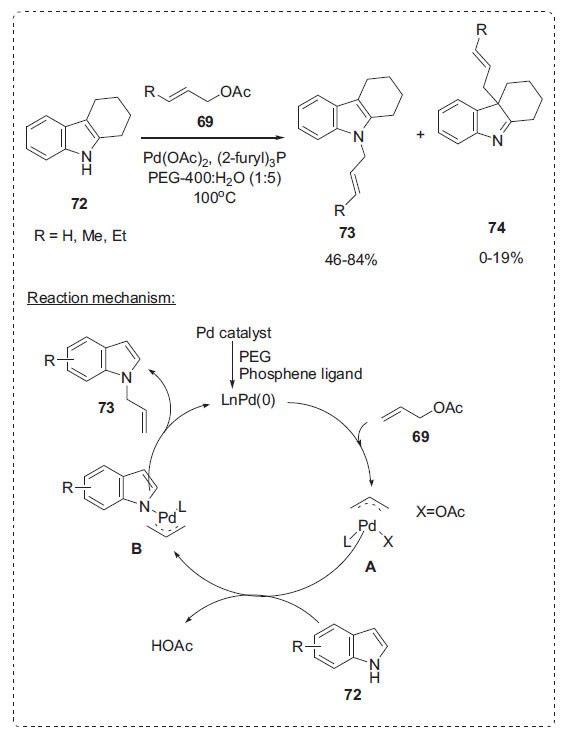
Selective *N*-allylation of indoles.

As a follow-up, Peng *et al*. applied allylic acetates **69** for the allylation of indoles **72** to prepare *N*-allylic indoles **73**, **74** *via* palladium-catalyzed transition metal as a promoting agent in PEG-H_2_O solvent medium (Scheme **22**) [89]. This technique offers a straightforward, convenient, and effective method for producing a high yield of *N*-allylated indoles. A plausible reaction mechanism for the synthesis of **73** from indoles **72** is illustrated in Scheme **22**. Initially, the PEG and phosphine ligand promote palladium catalyst to generate LnPd(0) which participate in the next circulation pathway. The entire circulation pathway consists of three steps: allyl acetate **69** with LnPd(0), allylation of the indole, and elimination from the *π*- allylpalladium intermediate. As detailed, **69** reacts with Pd(0) and the phosphine ligand species, which generate *in situ*, to produce *π*-allylpalladium intermediate **A**. Subsequently, the *π*-allylpalladium intermediate with indole **B** is followed by the allylation of **A** with indole **72**. Finally, the elimination of the *π*-allylpalladium intermediate with indole **B** establishes the C-N bond formation. Then, the whole system gives *N*-allylation of indole **73**.

### 2.10 Cyanation

**Scheme 23:**
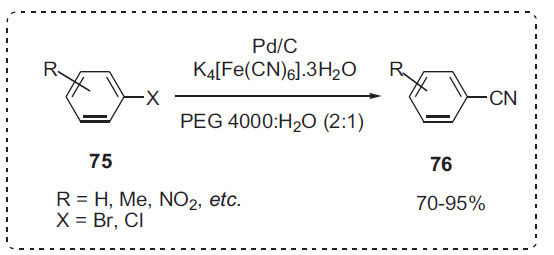
Cyanation of aryl halides.

One of the most convenient methods for preparing aryl nitriles is transition-metal-mediated cyanation of aryl halides. Chen *et al*. developed an environmentally friendly Pd/C-PEG-H_2_O system for the cyanation of aryl halides under microwave irradiation (Scheme **23**) [90]. A variety of aryl bromides, iodides, and activated chlorides **75** were cyanated to compounds **76** utilizing the nontoxic K_4_[Fe(CN)_6_]·3H_2_O. This approach uses no phosphorus or nitrogen ligands or solvents. Furthermore, this reaction can be performed without the need for an inert environment. The findings revealed that the correct amount of water was important to the reaction's success. The yield improved considerably when the mass ratio of PEG 4000 to water was 2:1. PEGs were used as both polar co-solvents and PTCs in this process.

### 2.11 Fluorination

**Scheme 24:**
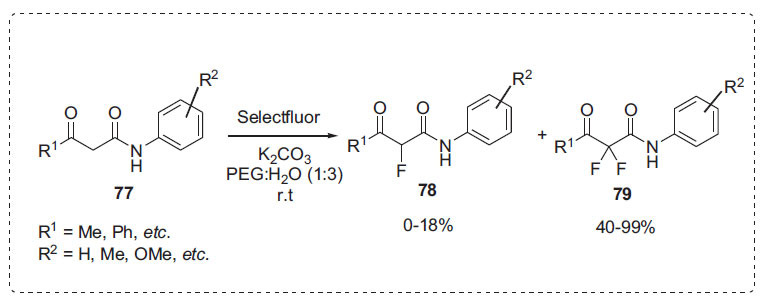
Synthesis of *α*,*α*-difluoro-*β*-ketoamides.

Zhang *et al*. reported the electrophilic fluorination of acetoacetamides **77** for the synthesis of *α*-fluoro-*β*-ketoamides **78** and *α*,*α*-difluoro-*β*-ketoamides **79** by using Selectfluor as the F^+^ source in the presence of K_2_CO_3_ in the mixed-solvent of PEG-400 and water and in the ratio of 1 to 3 (Scheme **24**) [91]. This approach does not require the use of an additional phase-transfer catalyst or tedious work-up. Using this method, the majority of examples investigated in this work resulted in nearly quantitative conversions, independent of the electronic nature of the substituent pattern.

### 2.12 Selenylation

**Scheme 25:**
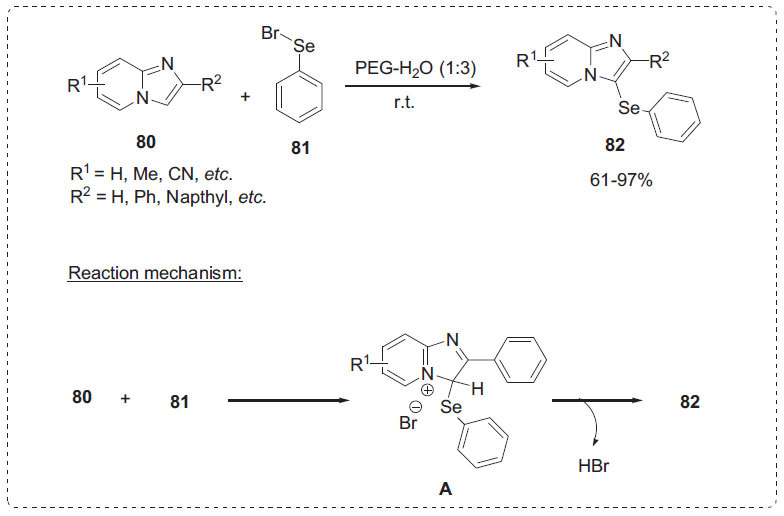
Selenation of imidazo [1,2-*a*]pyridine.

Organoselenium compounds have gained a lot of attention because they are found in a wide variety of molecules that have a variety of biological activities [92]. Jana *et al*. developed a viable method for electrophilic substitution phenylselenylation of imidazo [1,2-*a*]pyridines **80** in PEG-H_2_O media at room temperature using phenylselenium bromide **81** (Scheme **25**) [93]. The best result was obtained in a mixture of PEG-H_2_O (1:3) solvent system. This approach was used to synthesize a library of 3-phenylselenylimidazo [1,2-*a*]pyridines **82** with good yields. The current methodology is applicable to gram-scale synthesis without a significant decrease in yield. This method is also applicable for the phenylselenylation of imidazo [2,1-*b*]thiazole and benzo[*d*]imidazo [2,1-*b*]thiazole. Initially, the electrophilic addition of PhSeBr **81** occurs at the 3-position of imidazopyridine moiety **80** to produce the imidazolenium intermediate **A**. Finally, intermediate **A** produces product **82** through the elimination of HBr.

### 2.13 Amination

**Scheme 26:**
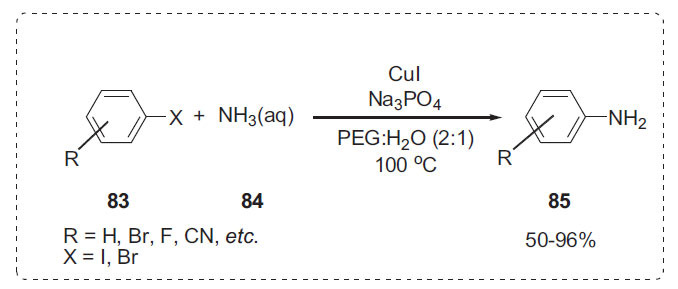
Amination of aryl halides.

A high-yielding cross-coupling reaction between aryl halides **83** and aqueous ammonia **84** was described by Chen *et al.* using the CuI/PEG-400 system (Scheme **26**) [28]. A wide range of electron-withdrawing or electron-donating aryl iodides and bromides were found to be applicable to the solvent system to produce aromatic amines **85**. The procedure allows for the assembly of a wide range of primary arylamines with functional groups such as cyano, nitro, acetyl, ether, or amino moiety. In the current reaction system, PEG-400 was used not only as a solvent but also as a phase transfer catalyst.

## 3 SYNTHESIS OF HETEROCYCLIC COMPOUNDS

### 3.1 Five and Six Membered Heterocyclic Compounds

#### 3.1.1 Triazoles

**Scheme 27:**
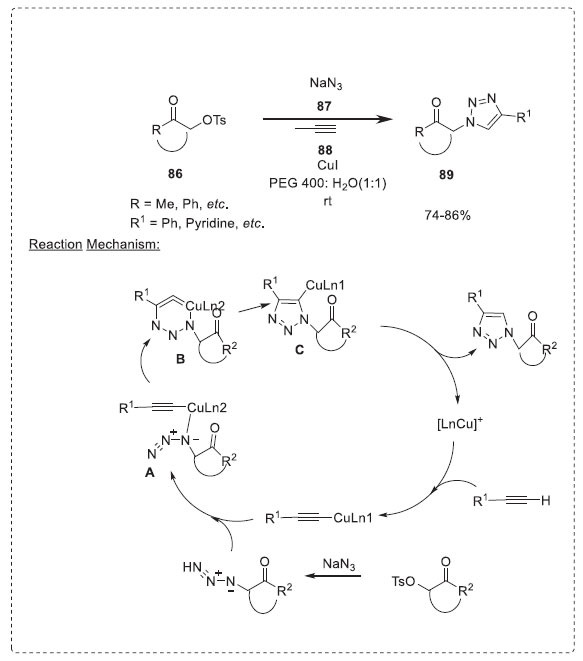
Synthesis of 1,4-disubstituted triazoles.

1,2,3-triazole are useful building blocks and are reported to have several biological activities [94]. In this regard, Kumar *et al*. described a reaction *α*-tosyloxy ketones **86**, sodium azide **87**, and terminal alkynes **88** in the presence of copper(I) in aqueous polyethylene glycol to produce regioselectively 1,4-disubstituted 1,2,3-triazoles **89** in good yield at room temperature (Scheme **27**) [95]. The authors verified various solvent system combinations before choosing PEG 400/H_2_O (1:1) to achieve the best results. They also observed that the reaction time with this aqueous PEG 400 system was as low when compared to with *t*-BuOH/H_2_O due to the phase-transfer catalytic nature of PEG. The one-pot exclusive formation of 1,4-disubstituted 1,2,3-triazoles involves *in situ* formation of *α*-azido ketones, followed by cycloaddition reaction with terminal alkyne. The versatility of this one-pot technique was illustrated by the synthesis of a variety of 1,4-disubstituted 1,2,3-triazoles. The use of copper(I) as a reusable catalyst in aqueous PEG simplifies, reduces costs, and is environmentally benign. The step-wise mechanism involves the initial formation of the *α*-azido ketone from the reaction of *α*-tosyloxy ketone **86** with sodium azide. The copper(I) quickly forms an acetylide with the terminal alkyne, which in turn forms adduct **A** with *α*-azido ketone. Subsequent intramolecular cyclization of **A** produces another cyclic adduct **B,** which rearranges to copper-containing 1,2,3-triazole **C**. Finally, the protonation of **C** leads to 1,2,3-triazole **89** and catalyst regeneration.

**Scheme 28:**
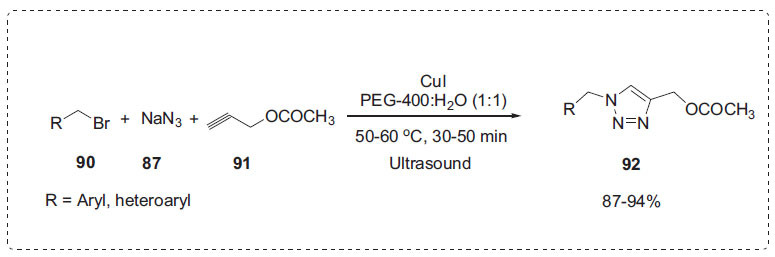
Synthesis of triazoles.

Inspired by this work, Reddivari *et al*. developed another method for the direct synthesis of 1,4-disubstituted triazole derivatives **92** in the presence of PEG-400:H_2_O (1:1) medium through a “click reaction” of aryl bromides **90**, sodium azide **87** and alkyne **91** promoted by ultrasonic radiation (Scheme **28**) [96]. This approach uses azide-alkyne [3+^2^] cycloaddition, which is catalyzed by copper (I). When compared to other co-solvent systems and individual solvents, the authors observed that the reaction was most successfully carried out in PEG-400:H_2_O (1:1) solvent system. When the water concentration in the solvent solution was increased, the yield of the product decreased slightly. The green solvent system employed has been efficaciously reused several times without any loss of its activity.

**Scheme 29:**
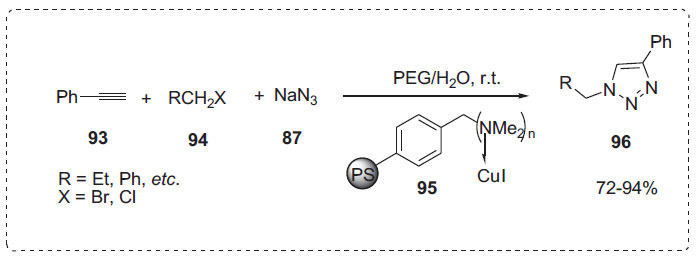
Synthesis of 1,4-disubstituted 1-alkyl-1,2,3-triazoles.

Similarly, Wang *et al.* reported a one-pot synthesis of 1,4-disubstituted 1-alkyl- and 1-aryl-1,2,3-triazoles **96** *via* 1,3-dipolar cycloaddition of alkyl halides **94**, sodium azide **87**, and phenylacetylene **93** using polymer-supported catalyst **95** (Scheme **29**) [97]. The reactions proceeded easily at room temperature using PEG-H_2_O as the solvent, yielding triazoles **96** in good to excellent yields. PEG 400/H_2_O (1:1) was determined to be the best solvent after the authors evaluated various solvent system combinations. This outcome was attributed to the phase-transfer catalytic properties of PEG 400 and the possibility that water could improve sodium azide's solubility. The catalyst was easily recoverable through filtration and could be recycled multiple times with only a minor loss in activity.

**Scheme 30:**
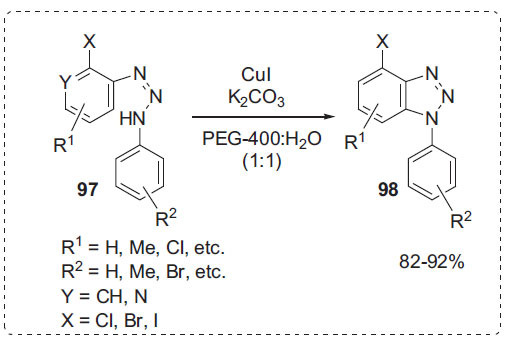
Synthesis of benzotriazoles.

Mukhopadhyay *et al*. reported another method for the synthesis of different regiospecific 1-arylbenzotriazoles **97** *via* intramolecular *N*-arylation of diazoaminobenzenes of 2-haloaryldiazonium salts **98** in PEG-water (Scheme **30**) [98]. Using PEG-400 in water, the desired product was obtained in excellent yield which was even better than in DMF or in DMSO. PEG acts as a phase transfer catalyst and increases the solubility of the diazoaminobenzenes in aqueous medium. The key characteristics of this protocol include mild reaction conditions, a vast number of affordable substrates, and great yields.

#### 3.1.2 Thiazoles

**Scheme 31:**
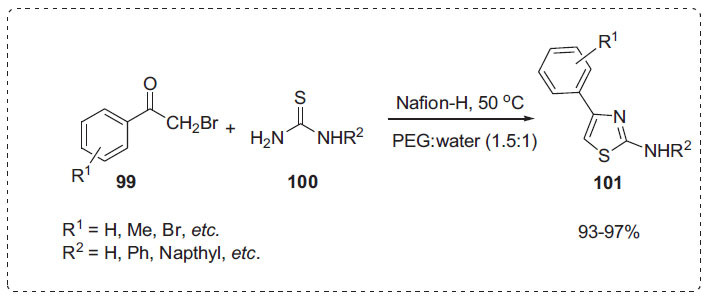
Synthesis of 2-aminothiazoles.

Thiazoles are biologically active and highly desirable in medicinal chemistry [99]. Using a PEG-H_2_O solvent combination, Kidwai *et al.* devised a process for the synthesis of 2-aminothiazoles **101** by the reaction of phenacyl bromide **99** and thiourea **100** in the presence of catalytic amount of Nafion-H (Scheme **31**) [100]. The authors examined the optimal reaction conditions by varying the PEG-H_2_O solvent system ratios. They discovered that reducing the amount of PEG from 100% to 60% enhanced the product yield slightly, but further reducing the ratio of PEG lowered the product yield, which is linked to the reactant's loss of solubility. This technique is an appealing, environmentally acceptable synthetic tool for the synthesis of these substrates due to the heterogeneous nature of the catalyst, its simplicity/reuse, excellent product yields, and ease of work-up.

#### 3.1.3 Pyrroles

**Scheme 32:**
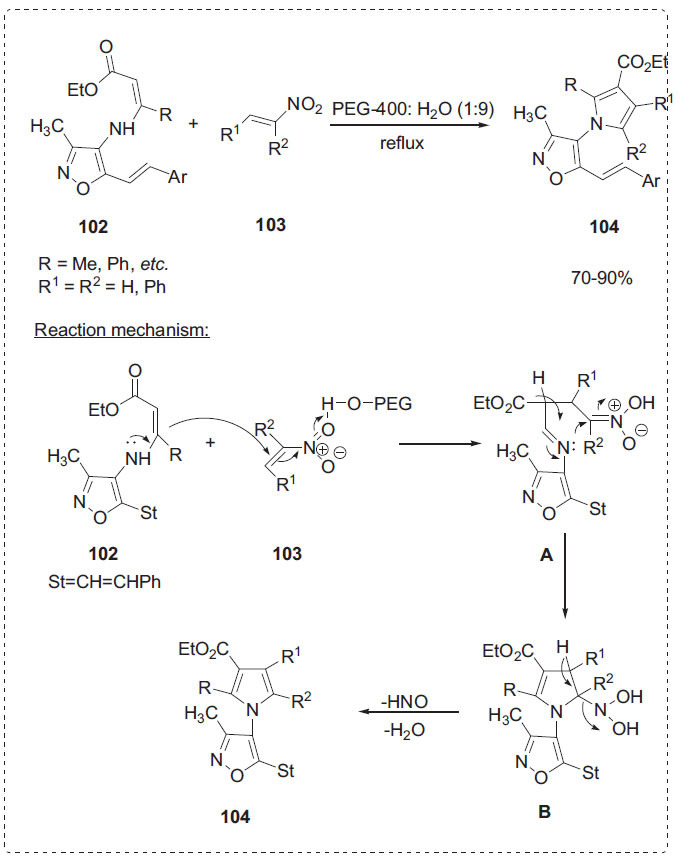
Synthesis of isoxazole substituted pyrroles.

Pyrrole is well known as a biologically active scaffold that possesses a diverse nature of activities [101]. To synthesize new isoxazolyl pyrrole derivatives **104**, Ponduri *et al*. reported a highly improved protocol from a one-pot reaction of isoxazolyl enamino esters **102** and nitro olefins **103** by using PEG-400/H_2_O under metal-free conditions (Scheme **32**) [102]. The authors examined the influence of other solvents such as THF, EtOH, DMF, CH_3_CN and DMF in the presence of PEG-400 under reflux conditions and found that PEG-400 water mixture was the best catalyst reaction medium for this one-pot reaction. After completion of the reaction, PEG-400 was recovered and reused in five consecutive runs without much loss of efficiency. A plausible mechanism for the formation of isoxazolyl pyrroles **104** is proposed in Scheme **32**. Here, PEG-400 acts as a Lewis acid catalyst. In the first step, the conjugate addition of compound **102** to the **103** in the presence of PEG-400 to give the Michael adduct **A**. Then, intermediate **A** was instantly attacked by the nitrogen atom to form the intermediate **B**. Finally, there elimination of water and nitroxyl molecules to afford the desired product **104** (Scheme **32**).

#### 3.1.4 Indoles

**Scheme 33:**
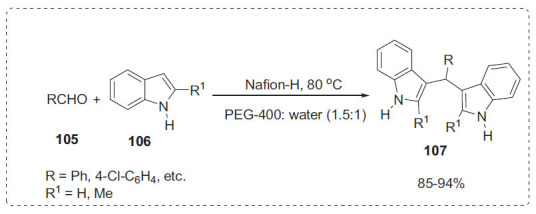
Synthesis of bis(indolyl)methanes.

Indoles and their derivatives are widely recognised as an important class of heterocyclic compounds [103]. In the PEG-400:H_2_O solvent solution, Kidwai *et al*. have demonstrated Nafion-H^®^ as an efficient and eco-friendly catalyst for the synthesis of bis(indolyl)methanes **107** from electrophilic substitution of indoles **106** with aromatic aldehydes **105** (Scheme **33**) [104]. The authors examined various solvent system and found that PEG: H_2_O (1.5:1) was the best solvent system for this reaction. The catalyst may be retrieved and reused numerous times after the reaction is completed with no substantial loss in catalytic potential. This methodology significantly improved the synthesis of bis(indolyl)methanes in terms of product yield, operational simplicity, and environmental issues by avoiding the use of expensive, hazardous catalysts and solvents.

### 3.2 Fused Heterocyclic Compounds

**Scheme 34:**
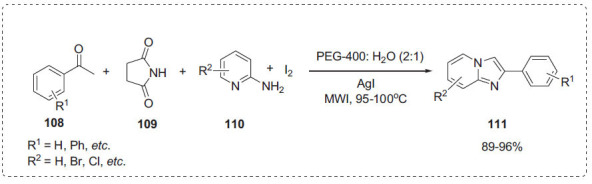
Synthesis of 2-phenylimidazo [1,2-*a*]pyridines.

Jadhav *et al*. developed synthesis of 2-phenylimidazo [1,2-*a*]pyridines **111** by a one-pot reaction of acetophenones **108**, succinamide **109**, 2-aminopyridine **110** and iodine with green solvent PEG-400 and water under microwave irradiation (Scheme **34**) [105]. The authors found that a mixture of PEG and water (2:1) increased the product yield in less time. This could be attributed to the hydrophilic and thermodynamically more favourable nature of PEG solvent. The advantage of this method is the newly developed protocol, which produces a high yield of product in a short period of time, avoiding the usage of lachrymatric *α*-chloro and *α*-bromocarbonyl compounds, volatile, poisonous organic and hazardous solvents and reagents.

**Scheme 35:**
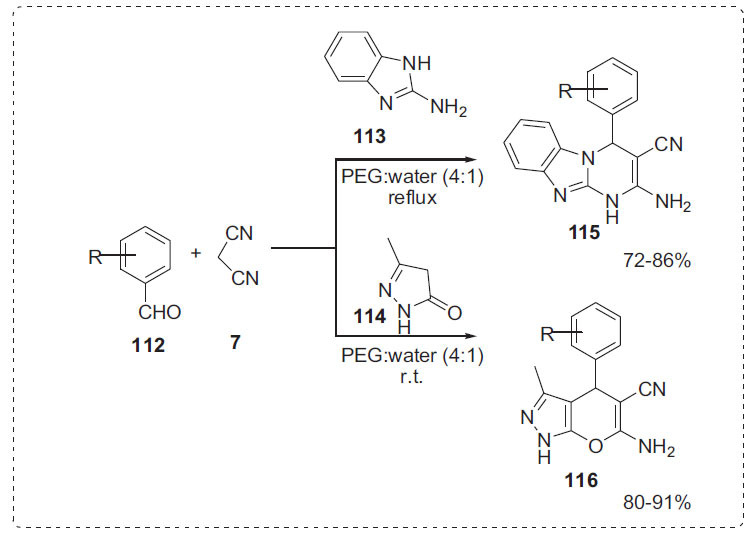
Synthesis of pyrimido [1,2-*a*]benzimidazole and pyrano [2,3-*c*]pyrazole derivatives.

Similarly, Survase *et al*. reported the synthesis of pyrimido [1,2-*a*]benzimidazole **115** and pyrano [2,3-*c*]pyrazole **116** derivatives by the reaction of aldehydes **112**, malononitrile **7** with 2-aminobenimidazole **113** or 3-methyl-1*H*-pyrazol-5(4*H*)-one using polyethylene glycol (PEG-400) in water under catalyst-free conditions (Scheme **35**) [106]. Because knoevenagel condensation takes longer in PEG 400, the authors discovered that only a modest amount of water in PEG is required to catalyze knoevenagel condensation at a higher rate. The greatest results in PEG-H_2_O (4:1) were obtained by optimizing reaction conditions at varied PEG: H_2_O ratios. Almost all products were insoluble in PEG solution, so as the reaction progresses, compounds precipitate slowly from the reaction mixture. Because of PEG's strong hydrophilicity, water might be used to separate the products from the reaction mixture. Following the reaction, the polyethylene glycol was easily isolated from the reaction media and distilled under vacuum to recover solvent for future reactions. After three successive runs, recycled PEG showed no loss of efficiency with regard to reaction time and yield.

**Scheme 36:**
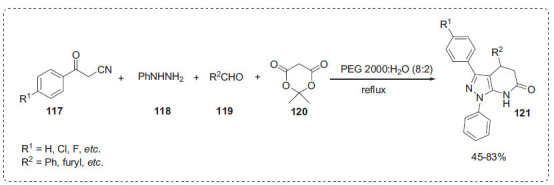
Synthesis of pyrazolo [3,4-*b*]pyridinones.

Using a PEG-H_2_O mixed solvent medium, Zeng *et al*. developed a novel protocol for the synthesis of 6,7-aromatic substituted pyrazolopyridine derivatives **121** from substituted benzoyl acetonitrils **117**, 1-phenylhydrazine **118**, substituted benzaldehydes **119** and Meldrum acid **120** in a four-component one-pot reaction without any catalyst (Scheme **36**) [107]. It was proved that water was an important promoter of the reaction and PEG 2000 was found to improve the reaction in terms of yield. Water was shown to be a major promoter of the reaction, and PEG 2000 was discovered to increase the yield. The PEG-H_2_O reaction media not only aided the reaction but also made separation easier. PEG 2000-H_2_O reaction media was effectively recycled and reused at least 5 times with no noticeable yield reduction.

**Scheme 37:**
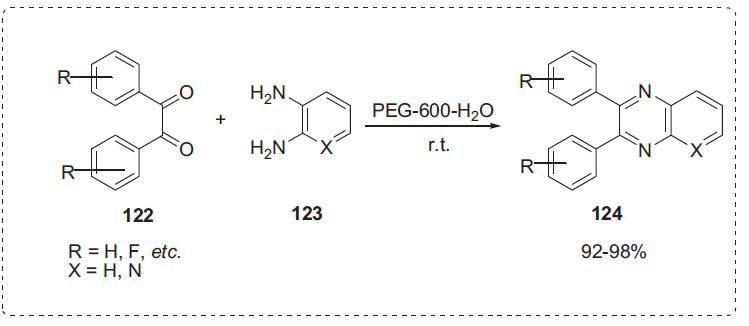
Synthesis of quinoxaline derivatives.

Chavan *et al*. demonstrated a protocol for the synthesis of quinoxalines **124** from 1,2-diketones **122** and 1,2-diamines **123** using PEG-600 and water mixture solvent medium (Scheme **37**) [108]. This low-cost, non-toxic, environmentally safe, and easily accessible approach successfully condensed numerous aromatic and aliphatic 1,2-diketones **122** with aromatic and aliphatic 1,2-diamines **123**, yielding the corresponding products in good yields. The addition of water to PEG increased the rate of the reaction and the yield. The maximum reaction rate was observed when the PEG-H_2_O proportion was 1:1.

**Scheme 38:**
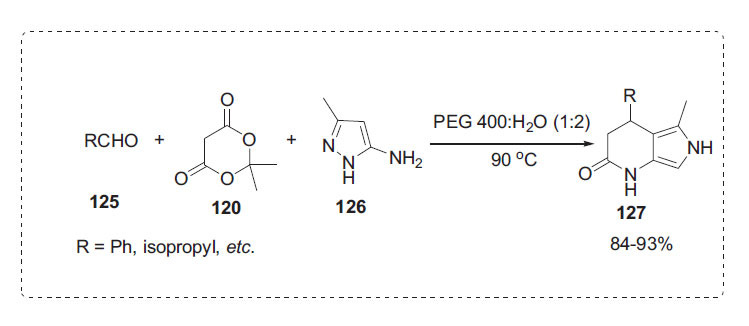
Synthesis of pyrazolopyridine derivatives.

Zhong *et al*. reported the synthesis of pyrazolo [3,4-*b*]pyridine-6(7*H*)-one derivatives **127** in a three-component reaction involving an aldehyde **125**, Meldrum's acid **120**, and 3-methyl-1*H*-pyrazol-5-amine **126** using recyclable polyethylene glycol PEG-400 in water (Scheme **38**) [22]. This technique is renowned for its environmental acceptability, high yields, ease of set-up, cleaner reaction profiles, environmentally friendly solvent, and PEG recyclability.

**Scheme 39:**
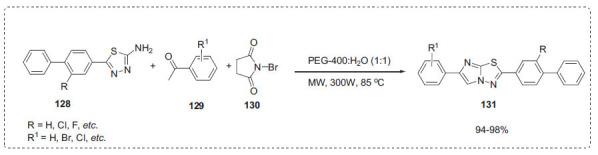
Synthesis of biphenylimidazo [2,1,-*b*][1,3,4]thiadiazole derivatives.

Wagare *et al*. reported a simple, rapid, green, and one-pot cascade process for the synthesis of benzo[*d*]imidazo [2,1-*b*]thiazoles **131** *via* microwave irradiation at 80-85°C in PEG-400 and water as a green reaction medium from the condensation of aromatic ketones **129**, NBS (*N*-bromosuccinimide) **130**, and -(biphenyl-4-yl)-1,3,4-thiadiazol-2-amine **128** (Scheme **39**) [109]. When compared to 1:2, 1:3, 1:4, and PEG alone, PEG-400/water at a 1:1 proportion offered the highest yield. In a short amount of time, the products were obtained in good to excellent yields.

**Scheme 40:**
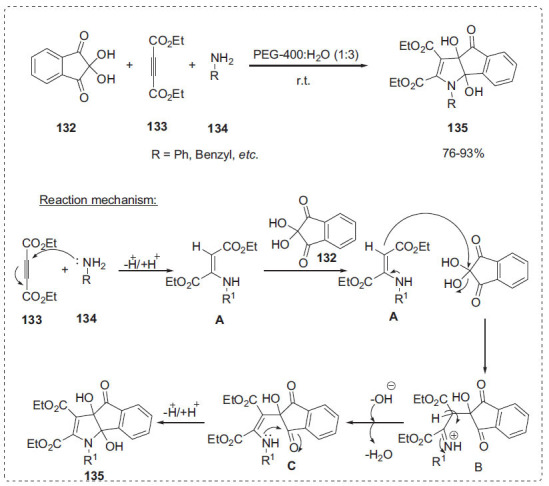
Synthesis of dihydroindeno [1,2-*b*]pyrrole derivatives.

Mal *et al*. reported a simple and environmentally friendly reaction technique for the synthesis of new dihydroindeno [1,2-*b*]pyrroles **135** as chemosensors in good yields using a three-component coupling reaction of diethylacetylenedicarboxylate **133**, amines **134**, and ninhydrin **132** in PEG-H_2_O (Scheme **40**) [110]. The product yield was less when the reaction was performed in PEG-400, but a considerable improvement in yield was found when the reaction was performed in PEG-400/H_2_O in a 1:3 (v/v) ratio. This is due to PEG's role as a phase transition catalyst. PEG-400 was shown to be the optimum solvent for the reaction among the numerous PEGs tested. The important benefits of this technique include the fact that the reaction occurs at room temperature in a short reaction time and with simple workup processes, resulting in good yields. Initially, amine **134** reacts with dialkylacetylenedicarboxylate **133** to form an enaminediester intermediate **A**. Then the, intermediate **A**, which acts as a nucleophile, attacks the more electrophilic carbonyl centre of ninhydrin **132** to produce intermediate **B**. After that, intermediate **B** is converted to intermediate **C** with the removal of a water molecule. Finally, intramolecular cyclization of intermediate **C** with a tautomerization step resulted in the ultimate target compound **135**.

**Scheme 41:**
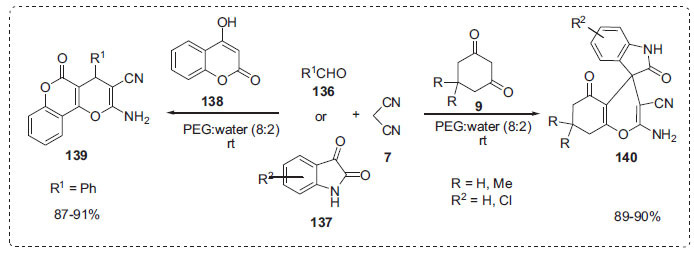
Synthesis of 4*H*-pyran derivatives.

Survase *et al*. developed a green, one-pot, multicomponent method for the synthesis of a diverse library of 4*H*-pyran derivatives, including spirochromenes **140** and dihydropyrano [3,2-*c*]chromines **139**, by reacting aldehydes **136** or isatins **137** with 4-hydroxy-2*H*-chromen-2-one **138** or dimedones **9** in water with PEG-600 as a promoting reaction medium (Scheme **41**) [111]. The PEG-H_2_O (8:2) solvent system produced the good results. Because of PEG's strong hydrophilicity, water might be used to separate the products from the reaction mixture. As the reaction progressed, the resultant precipitate steadily separated from the reaction mixture. PEG-H_2_O as a solvent solution not only simplifies product separation but also improves environmental compatibility and sustainability due to the elimination of quenching stages, decreased reliance on harmful organic solvents, and waste minimization.

**Scheme 42:**
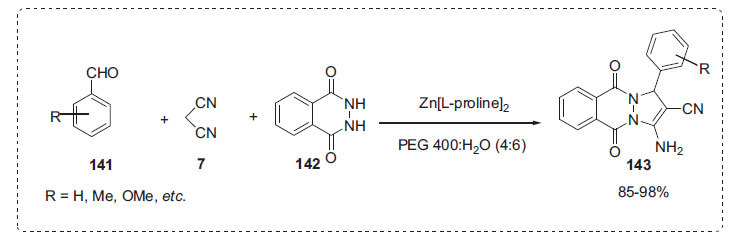
Synthesis of 1*H*-pyrazo [1,2-*b*]phthalazine-5,10-dione derivatives.

Yan *et al*. then developed a simple, efficient, and environmentally friendly method for synthesizing 1*H*-pyrazolo [1,2-*b*]phthalazine-5,10-diones **143** using a one-pot three-component reaction of aromatic aldehyde **141**, malononitrile **7**, and phthalhydrazide **142** catalysed by zinc-proline complex (Zn[L-proline]_2_) in PEG 400:H_2_O solvent (Scheme **42**) [112]. The model reaction was performed in many solvents at 80°C in the presence of Zn[L-proline]_2,_ and it was discovered that the combined solvent of PEG 400 and water (4:6) as a green solvent produced the best results. This method has a number of advantages, including a straightforward operation technique, gentle reaction conditions, an ecologically friendly catalyst, and better atom economy.

## 4 OTHER ORGANIC REACTIONS

**Scheme 43:**
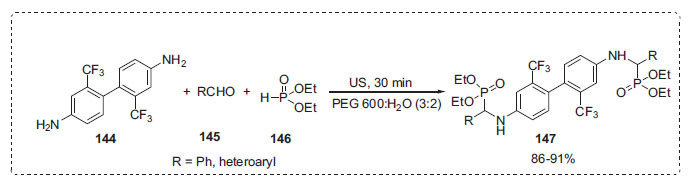
Synthesis of di-*α*-aminophosphate derivatives.

Uma *et al*. synthesized a variety of new di-*α*-aminophosphonate derivatives **147** in a one-pot process involving 2,2'-di(trifluoromethyl)biphenyl-4,4'-diamine **144**, different substituted aldehydes **145**, and diethyl phosphite **146** in the presence of PEG-H_2_O and ultrasonic irradiation (Scheme **43**) [113]. The current synthetic protocol's key advantages are its mild, solvent-free, reusable, ecofriendly catalyst and simple reaction work-up technique.

**Scheme 44:**
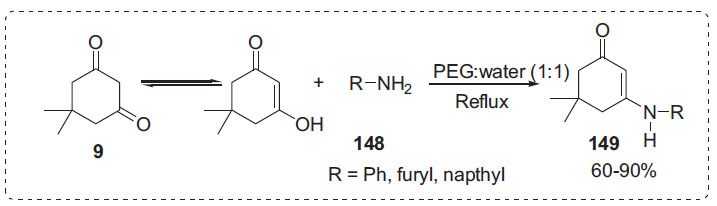
Synthesis of *β*-enamino ketones.

Bandgar *et al*. created *β*-enamino ketones **149** by reacting aromatic or aliphatic amines **148** with 1,3-dicarbonyl compounds **9** in polyethylene glycol (PEG)-600-water (Scheme **44**) [114]. The technique does not necessitate the inclusion of a catalyst or the azeotropic removal of water. The procedure can also be used to synthesize *β*-enamino esters. Furthermore, PEG-600 can be recycled multiple times and recovered in nearly pure form by distilling the aqueous layer under vacuum.

**Scheme 45:**
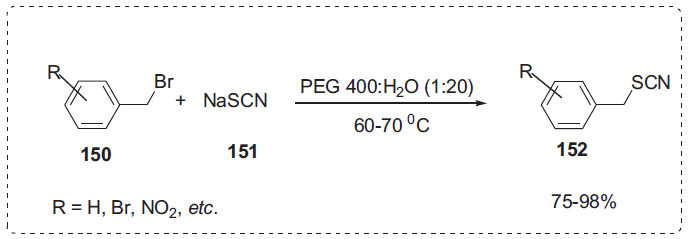
Synthesis of alkyl thiocyanates.

Awasthi *et al*. reported a microwave-assisted reaction of alkyl bromide **150** and sodium thiocyanate **151** to produce alkyl thiocyanates **152** in the presence of PEG-H_2_O as a safe reaction medium (Scheme **45**) [115]. After completion of the reaction, the pure product precipitates out of the reaction medium on cooling and is collected by vacuum filtration. The solvent system was reused continuously in repeated runs by the same procedure for the preparation of representative alkyl thiocyanate.

## CONCLUSION

PEG has been used widely in the synthesis of organic compounds as a phase-transfer catalyst and reusable reaction media. Its characteristics make it less environmentally damaging than other organic solvents. Numerous reactions, including named reactions, cross-coupling reactions, C-H activation reactions, hydrogenation, cyanation, *etc*., have been investigated in the PEG-H_2_O mixed solvent system. Additionally, a lot of heterocyclic scaffolds were synthesized utilizing the PEG-H_2_O solvent media. PEG participates in some of these reactions as a solvent and in others as a phase transfer catalyst. Different PEG-H_2_O solvent system ratios were studied for these processes. It was found that mixed PEG-H_2_O solvent solutions outperformed PEG or water alone. This solvent media improves the reactants' solubility in water while also speeding up the reaction with great yields. Because quenching phases are eliminated, toxic organic solvents are used less frequently, and waste is minimized, using PEG-H_2_O as a solvent solution enhances environmental compatibility and sustainability. The catalyst may be easily separated and reused, which is another benefit of this environmentally friendly reaction medium. Both the solvent solution and the catalyst are frequently reused in reactions with no appreciable activity loss. Additionally, this solvent media is appropriate for large-scale synthesis in the pharmaceutical industry due to its low toxicity and biodegradability.

As a result of the preceding discussion, it is worth noting that there is ample scope in the sphere of PEG-H_2_O, so existing conventional technologies as well as alternative processes, particularly those that are environmentally friendly and renewable and show high impact in the near future, should be explored. It is hoped that this review would spark interest in expanding PEG-H_2_O applications to new catalytic processes. The use of PEG-H_2_O in synthetic organic chemistry is both an opportunity and a difficulty, and the widespread availability of PEG at low cost may provide such procedures a promising future in green chemistry and green engineering. In this context, it is expected that research in this sector will become even more prevalent in the near future, notably in the fields of renewable energy resources and high-value chemical synthesis. It is expected that research in this area will expand in the near future, notably in the areas of multicomponent reactions and heterocyclic molecule synthesis.

## LIST OF ABBREVIATIONS

DESs: Deep Eutectic Solvents
ILs: Ionic Liquids
MCRs: Multi-component Reactions
PEG: Polyethylene Glycol
